# Experience of oncology residents with death: a qualitative study in Mexico

**DOI:** 10.1186/s12910-019-0432-4

**Published:** 2019-12-05

**Authors:** Asunción Álvarez-del-Río, Edwin Ortega-García, Luis Oñate-Ocaña, Ingrid Vargas-Huicochea

**Affiliations:** 1 Departamento de Psiquiatría y Salud Mental, Facultad de Medicina, Universidad Nacional Autónoma de México, Mexico City, Mexico; 2 Internal Medicine Resident, Departamento de Psiquiatría y Salud Mental, Facultad de Medicina, Universidad Nacional Autónoma de México, Mexico City, Mexico; 3 Surgical Oncology Consultant, Subdirección de Investigación Clínica, Instituto Nacional de Cancerología, Mexico City, Mexico; 4 Oficina 6 de la Coordinación de Investigación del Departamento de Psiquiatría y Salud Mental, Edificio F de la Facultad de Medicina de la UNAM, Colonia Copilco Universidad, Circuito interior y Cerro del Agua s/n, Ciudad Universitaria, Coyoacán, 04510 Ciudad de México, Mexico

**Keywords:** Physician-patient relations, Attitude to death, Truth disclosure, End of life care

## Abstract

**Background:**

Physicians play a fundamental role in the care of patients at the end of life that includes knowing how to accompany patients, alleviate their suffering and inform them about their situation. However, in reality, doctors are part of this society that is reticent to face death and lack the proper education to manage it in their clinical practice. The objective of this study was to explore the residents’ concepts of death and related aspects, their reactions and actions in situations pertaining to death in their practice, and their perceptions about existing and necessary training conditions.

**Methods:**

A qualitative approach was used to examine these points in depth based on interviews conducted with seven oncology residents.

**Results:**

Participants do not have a clear concept of death and, although it is seen as a common phenomenon, they consider it an enemy to beat. The situations to which respondents react more frequently with frustration and sadness after the death of patients were when they felt emotionally involved, if they identify with the patient, in cases of pediatric patients and with patients who refuse treatment. To deal with death, participants raise barriers and attempt to become insensitive. Although residents in this study recognize the importance of training to learn how to better deal with death, it seems they are not fully invested in reaching more of it.

**Conclusions:**

Participants face death in a daily basis without the necessary training, which appears to impact them more than they are willing to accept. They do not achieve their goals managing situations regarding death as well as they assume they do. Despite recognizing the need of more training and support for better coping with death, they prefer to continue to learn from their experience.

**Trial registration:**

Not applicable.

## Background

Although we are all aware that death is a part of life, an attitude of denial and silence currently prevails and prevents us from preparing to deal with the last stage of life [1]. Death can occur suddenly, but in most cases, it results from disease or a chronic condition that usually requires medical care and therefore, takes place in a medical context [2]. Physicians are compelled to prepare themselves to face death in their clinical practice. This requires early reflection on their own mortality and that of their close ones. Physicians must be capable of accompanying their patients, alleviate their suffering and worries, and clearly inform them on their personal situation, so that patients can decide in a timely manner what steps to follow in these last stages of life. Unfortunately, in reality, physicians are as reticent to face death as the rest of society and are improperly and insufficiently trained to manage terminal situations in their clinical practice [3]. Many find it difficult to provide adequate care to terminally ill patients, and experience their death as a personal failure, with a halo of guilt [4, 5]. This explains why they often continue to provide treatments that no longer benefit patients, but only prolong their agony. Further, they are reluctant to openly discuss the situation with them [6–8].

These points have been underscored in different international journals, proving that this is a global phenomenon. In the United Kingdom, a survey of 1003 general practitioners revealed that one in four physicians had never broached the subject of end-of-life (EOL) choices with their patients, although an average of 20 patients per general practitioner succumb every year. This study also revealed that physicians themselves feel uncomfortable fathoming their own death, as only 33% reported have spoken to their families about their EOL wishes and only 57% had a written will [9]. This has significant repercussions on patients, many in a terminal stage, who remain unaware of their alternatives in the last moments of their lives. A study by Hedland et al. [10] reported that 76% of interviewed patients had thought about their EOL decisions, but only 30% had discussed them with their family physician. Notably, only 11% of patients chose the option of life-prolonging therapy. Kale et al. [11] conducted the National Health and Aging Trends Study (NHATS), revealing that 40% of the interviewed population had not discussed their preferences in terms of EOL care. These two publications underscore the difficulty that physicians face when attempting to initiate this type of conversation with their patients. Finally, Boyd et al. [12], showed that these very necessary conversations tend to finally occur in the very last weeks of a patient’s life, once they have undergone several futile treatments and the need to receive timely palliative care is imminent.

Among all physicians, residents tend to be those who spend the greatest amount of time at the hospitalized patient’s bedside, including those who are terminal. However, their training is lacking in depth and breadth and they feel singularly unprepared to meet the patient’s needs [13]. It is important to establish what type of interactions exist between oncology residents and their patients, to identify the gaps that need to be filled and what aspects require feasible improvement [14]; further, the residents´ emotional needs in terms of anguish and anxiety should also be clearly addressed in order to determine what modifications could provide them with positive support [15].

Mexico is no exception in terms of the global lack of expertise in EOL care, including mastering the necessary and timely conversations with patients. Contrary to what one would think, although this is a country that celebrates and reveres the Dead with very attractive rituals and who even laughs at the concept of death, in reality and despite these traditions, most of the population does not discuss the matter with their family or peers and is rarely prepared to face their own mortality [16]. Lazcano et al. [17] suggest that an incomplete and vague communication pattern prevails between physicians and patients, resulting from the physician’s paternalistic attitude that tends to disregard patient autonomy. Another study [18], explored the attitude of Mexican physicians towards death in their practice, and found that only a minority of participants had received some form of training in the management of terminally ill patients (28%), but regardless, they still assumed their care (73%). Personal experience and years of practice had taught them how to face death, but still, most considered it would be wise and necessary to attend seminars or workshops on the subject (77%). In that study, most physicians asserted that patients should be informed when further curative treatments are of no use and that death is imminent; however, the information obtained did not allow the authors to determine whether the answers reflected the physicians´ actual beliefs or what they thought their responses should be, or their actual behavior when faced with terminal patients. In order to delve into the experience of oncology residents, physicians that frequently face death in their practice, we decided to conduct a study with a qualitative design, that has proven to be effective in better understanding personal and collective experiences and behaviors, taking into consideration environmental, social and cultural contexts that influence the way individuals deal with certain situations [19–21]. The aims of this project were to determine and analyze 1) the residents’ concepts on death and related aspects; 2) the residents´ reactions and actions in situations pertaining to death in their practice, and 3) the residents´ perceptions of the existing and necessary training conditions.

## Methods

This qualitative study followed an interpretative phenomenological approach to address the perceptions and experiences pertaining to death (as a life phenomenon and as a common event faced in Medicine) from the particular perspective of resident physicians in the medical area of Oncology. This study is compliant with the Consolidated Criteria for Reporting Qualitative Studies (COREQ) framework [22].

### Participants

Oncology residents training in a tertiary care Cancer Center in Mexico City were invited to participate in the study by one of the researchers (LFOO), an attending physician at the institution where the study was conducted. The invitation was open, the study and its objectives were communicated to all residents of the oncology areas and those who contacted LFOO to participate were included in the study. Sampling was purposive and non-probabilistic [23, 24]. The number of participants reflected the saturation of information, this means that recruitment ended when the ability to obtain new information had been attained [25]. In qualitative research, the sample size is small, since the main goal is to search for deep structural representation rather than statistical representativeness.

### Data collection

A semi-structured, focused or centered interview was used to explore the participants’ opinions and experiences. This type of interview combines the depth and flexibility of unstructured interviews with the administrative ease and clarity of structured interviews [21, 24]. In the interview process, several support tools are used, including an interview guide (available upon request) to probe key issues with the interviewees [23]. Table 1 shows the categories of analysis and examples of triggering questions. It is important to point out that the trigger questions are only examples of how to approach a topic since the interview guide is not a questionnaire; therefore, all the proposed questions are not asked because it depends on the rhythm of the interview with each participant.

**Table 1:** Categories of analysis and examples of triggering questions

Categories	Question examples
1. Concept of death (*the general and personal concept of death, the personal experiences associated with death and the concept of death in medical practice*)	What do you think about death? How would you define “death”? Have you ever had an experience with death, for example, a personal experience or with family/friends? Have you ever thought about your own death? Do you fear your death or that of others? As a physician, how do you face death? It is said that physicians have a tough attitude towards death, what do you think about that? As a physician, do you think that your obligation is to avoid death? Does the death of a patient make you think about your own death?
2. Actions and reactions toward death *(the recognition of imminent death in a patient, communication of imminent death to patients and families, reacting as a medical professional confronted with patient death, manners of coping with death and support to deal with death on a daily basis)*	How do you feel when you know that a patient has a terminal illness and death is inevitable? How do you decide whether you should inform the patient that he cannot be cured? Who do you inform first, the patient or the family? How do you tell a patient that death is inevitable? What is your attitude when a relative of your patient asks you to conceal information? Do you provide hope even if death is imminent? Do you think that there are other specialists that could better deliver bad news? Do you prefer to recommend other treatments instead of delivering bad news? How do you feel when one of your patients dies? Please describe to me any significant experience you have had dealing with the death of a patient
3. Training aspects to learn how to face death *(subjects such as the social representation of the physician figure, specific training on facing death as a physician, models and anti-models, teaching others to face death, the participants´ self-perceived ability to cope with death and their recognized needs)*	During your professional training, were you offered any academic formation on delivering bad news? What kind of reactions to death have you observed in your teachers? Among the actions and reactions around you, have any served as an example? Among the actions and reactions around you, have any seemed inappropriate or reprehensible? In terms of your professional formation, what do you believe is necessary to acquire more and better tools to face death? What would be your advice to a medical student or fellow resident?

Prior to the study, the interview guide was evaluated in a pilot study with three participants at the same hospital, in order to ensure the adequacy of the terminology for this population and the relevance of the established categories. The pilot study was also used to standardize the interview process between the two interviewers (a psychologist with a doctoral degree and significant experience in the subject of death (AAR), and a psychiatrist with a doctoral degree and solid knowledge of qualitative research (IVH).

### Procedures

Our local Ethics and Research committees evaluated and approved the protocol, as well as the study materials. The recruitment of participants began in 2012 and the interview guided was pilot-tested during 2012 and 2013. Throughout 2014 and 2015, one-on-one interviews were conducted with the participating oncology residents. AAR and IVH were the investigators responsible for conducting the interviews, digitally recording and transcribing them. Names and other identifying data were not included in the transcriptions to ensure confidentiality. During the interviews, participants were encouraged to speak freely and with the support of the interview guide, the topics relevant to the research were further delved into. In this study, the following categories were used in the final analysis: (1) Death, whereby the general and personal concept of death, the personal experiences associated with death and the concept of death in medical practice, were explored; (2) actions and reactions to death, which included the recognition of a patient’s imminent death, communication of imminent death to patients and families, reacting as a medical professional when confronted with patient death, manners of coping with death and support to deal with death on a daily basis; (3) training features focusing on strategies to face death, including subjects such as the social representation of the physician figure, specific training on facing death as a physician, models and anti-models, teaching others to face death, the participants´ self-perceived ability to cope with death and their recognized needs.

In general, the rhythm of the interviews was smooth and in moments in which there was some emotional reaction in the interviewee triggered by a memory or situation in particular, the interviewers could emotionally contain the person as stipulated in the ethical considerations of this study. At no time was refereeing (to a therapist or counsellor) necessary with a participant.

There were either one or two (in those cases in which the first interview could not be completed) interviews with each participant. The interviews lasted between 40 and 120 min each (on average 65 min). The participants were free to choose the most convenient time and place for the interview and they all opted for their hospital workplace. Interviews were conducted in an office that was a private, quiet and comfortable space.

### Analysis

Analysis of the transcribed interviews was performed with the technique of “meaning categorization” [20]. The transcripts were coded as a whole. Data were coded into mutually exclusive categories to facilitate the organization of long texts and analyze the information. Three researchers (AAR, EOG, and IVH) analyzed the transcriptions independently to discuss the findings and reach a common consensus.

To establish categories and subcategories, a mixed criterion was followed, that is, the procedure was both deductive and inductive. The starting point of the category development process was a deductive procedure, since this involved initially working with the themes extracted from the specific objectives of the project, and identifying themes based on the theory underlying the research. This deductive part constituted the skeleton of our categorization scheme. Once the work with the main categories was started, an inductive process was subsequently carried out, in which emerging categories were identified. The themes of these new categories were determined from the successive readings of each interview.

## Results

A total of seven (four men and three women) participants were included after voluntarily accepting to participate in this project. Their age ranged between 31 and 36 years, all were Mexican physicians, second or third-year residents, conducting their specialty in an area of the field of oncology: hematology, surgical oncology, medical oncology, radiation oncology. (Table 2).

**Table 2:** Demographic characteristics

Participants	Characteristics
Dr. 1	Female, resident in Hematology, Mexican, single.
Dr. 2	Male, resident in Surgical Oncology, Mexican, married with children.
Dr. 3	Male, resident in Medical Oncology, Mexican, married with children.
Dr. 4	Male, resident in Hematology, Mexican, single.
Dr. 5	Male, resident in Medical Oncology, Mexican, married with children.
Dr. 6	Female, resident in Hematology, Mexican, single.
Dr. 7	Male, resident in Radiation-Oncology, Mexican, married with children.

The information obtained was grouped into three main categories for analysis and presentation, including all the complementary subcategories (Table 1). The testimonies presented in this section are summarized in Table 3.

**Table 3:** Categories and subcategories of analysis and quotes

Categories	Example quotations by subcategories
1 Concept of death	1.1 General concept of death “[…] There is a chance that the spirit will go on, that is something very clear and very precise and that has been clear to me all my life […] a life ends and you do not know what there is beyond, right? […] “. (Dr.1) “[…] I obtained my concept at home: it’s based on my religion […]” . (Dr. 6)
	1.2 Personal concept of death “[...] At this moment, it would be something terrible [...] a person like me who has just graduated, just trained, I would say: ‘Death right now, ‘What horror’ [...] but in 30 years [...] it would be something that would not scare me […]” . (Dr. 3) “[…] I have lived so close to death that it’s not a stranger to me […] I’m not really afraid about my own death; what scares me is not knowing what would happen with my family […]” (Dr. 5)
	1.3 Personal experiences and reactions associated with death “[…] I had a tumor in the nasopharynx [...] I am very much blocking the tragic events in my life; I’ve always done so […] I remember that I was in the ICU [...] my parents crying everyday [...] it is not something that I reflect upon […].” (Dr. 2) “[…] I should have intervened, the symptoms were evident […] I still think about how the diagnosis was delayed [niece with leukemia] and I am very frustrated because it was something that could have had a different outcome […]” . (Dr. 6) “[...] and everyone is all over you: ‘Did you go see him?’ [...] And you think: ‘What should I say, what should I do, how do I get through this?’ I am not his doctor, so I cannot give a medical report […] I am his relative […]” . (Dr. 4)
	1.4 Concept of death in medical practice “[…] I don’t like to think that a patient doesn’t have a chance [...] I always offer something else that could be done […]” . (Dr. 3) “[…] I don’t like to say ‘there is nothing we can do’ […] it is a term that I avoid because you can almost always do something […] even if you can’t do something to eradicate the patient’s disease […] there is something that you can offer to alleviate the patient’s suffering and give him a better quality of life […]” . (Dr. 4)
2.Actions and reactions toward death	2.1 Recognition of imminent death in a patient “[…] There is a body of knowledge in the medical literature, then you can know how advanced the disease is and how likely it is for the patient to be cured [...] “. (Dr. 2) “[…] Over the years you realize that it is not only about medical aspects […] it is about how much the patient wants to live […] those who want to fight […] those who have family support […]” . (Dr. 5)
	2.2 Communication of imminent death to patients and families “[...] Something I always tell those patients that ask me ‘Am I going to die?’ is ‘Look, I would love to be the creator, to have a crystal ball so I could say Yes, the answer is yes, but I am human, I don’t know […] I can’t give you that answer’ […]” . (Dr. 7) “[…] You can’t ignore the relatives who are asking you not to do it [deliver bad news], but it seems to me that the patient has the right to know […] it makes me very angry that they are not told […]” . (Dr. 3) “[…] Well, we do not tell anyone as such that he is going to die. That is the advantage! […]” . (Dr. 6)
	2.3 Reacting as a medical professional to death “[…] I get really frustrated with those patients, then I get mad and say ‘Why don’t they want to try it if there is still something that can be done?’ […]” . (Dr. 1) “[…] I’ve done everything humanly possible for him […] I don’t feel frustrated because since one first begins to treat patients like these, one is aware that treatments have limitations […]” . (Dr. 5).
	2.4 Ways of coping with death. “[...] I do not want to relate too much with the patient [...] I frame a distance [...] is like my defense mechanism […]” . (Dr. 5) “[...] With my peers sometimes we joke about things related to diseases [...] so everything you live daily doesn’t be so overwhelming […]” . (Dr. 6) “[...] a very easy way out is to calmly establish limits and treat everyone as if they have a simple flu [...] I have not been able to do that [...] I’ve committed myself to the specialty […]” . (Dr. 1)
	2.5 Support to deal daily with death “[…] I talk to my wife; she is a physician; we talk about medical issues […]” . (Dr. 4) “[…] Buy things, read something, record music […] to diverge the tension […] it helps you to keep doing things better […]” . (Dr. 2)
3.Training aspects to learn how to face death	3.1 The social representation of the physician’s figure “[…] You study medicine to cure people […] your obligation is to help them as much as they want […]” . (Dr. 5) “[…] As a doctor, I put my thoughts and my energy into seeing what I can offer, in reasoning about suffering, not in living it […]” . (Dr. 1)
	3.2 Specific training to face death as a physician “[…] You see how they approach them [patients] and how the patient reacts [...] there was no one to sit and tell you how to do so, so you approach [to the teaching physicians] and you watch […]” . (Dr. 3) “[…] it’s something that is not learned, it is not something that is studied, it is something that is learned as you go […]” . (Dr. 2)
	3.3 Models and anti-models ”[…] He always said that they [patients] should be treated with respect and like we would want to be treated […]” . (Dr. 6) “[…] And he said: ‘look, of course you are bleeding, you have a lymphoma, do you know what that is? No, right? To stop the bleeding, I would have to get you to the operating room and I’m not going to do that now, so you will have to handle it’ […] that’s not the way! […]” . (Dr. 4)
	3.4Teaching others to face death “[…] I tried to teach them how to get close to the relatives […] I always try to show them how to do things […]” . (Dr. 3)
	3.5 Self-perceived ability to cope with death “[…] They never teach us how to deliver bad news […] I have no idea if my method is good, if it is bad or if it is worse, but it is the one that has worked for me […]” . (Dr. 1) “[…] Here, I see that the physicians in charge of the patient are not going to give the bad news; we, the residents, are the ones who generally have to do it […] the treating physician is not going to come here at 2 or 3 in the morning […]” . (Dr. 6) “[...] it is complicated if they aren’t my patients as it happens in a guard […] or when you receive a case in the emergency room and you have to inform the relatives that the patient is going to die; it is difficult because you don’t know them, you don’t know how they will react […]” . (Dr. 4)
	3.6 Perceived needs to cope with death “[…] Thinking about courses or groups or things like that, we don’t even have time, we are overworked, tired […] we live entire seasons in the hospital […] if you get one more class, what you think is ‘good, I’m going to sleep’[…]” . (Dr. 2) “… we say it jokingly, but we say that everyone in oncology has something wrong … I don’t know if it would be better if I had it [psychotherapy], or if I should look for it …” . (Dr. 7)

### Category 1. Concept of death

#### General concept of death

Participants did not have a clear concept of death; they conceived it as a transition in which the spirit continues to exist after separating from the body, although they were uncertain about what happens after death. The concept was acquired by beliefs originating in the religion that was inculcated at home.

*“[ … ] There is a chance that the spirit will go on, that is something very clear and very precise and that has been clear to me all my life [ … ] a life ends and you do not know what there is beyond, right? [ … ]“.* (Dr.1)

#### Personal concept of death

Most of the interviewees asserted that they do not fear their own death; they, however, are afraid to leave the people they love behind because they believe they will still be needed and will not be able to help them. In addition, dying now would be ill-timed since they have projects to conclude, and they prefer for death to occur in old age.

*"[...] At this moment, it would be something terrible [...] a person like me who has just graduated, just trained, I would say: 'Death right now, 'What horror' [...] but in 30 years [...] it would be something that would not scare me [ … ]".* (Dr. 3)

#### Personal experiences and reactions associated with death

The participants’ experiences with death had been limited to the death of relatives and in one resident’s case, with a very serious personal illness, but the prevailing attitude was to avoid the thought of ​​having been at risk of dying.

*“[ … ] I had a tumor in the nasopharynx [...] I am very much blocking the tragic events in my life; I’ve always done so [ … ] I remember that I was in the ICU [...] my parents crying everyday [...] it is not something that I reflect upon [ … ].”* (Dr. 2)

When faced with severe disease in their relatives, some participants wanted to be actively responsible for their treatment, adopting the role of the strong and knowledgeable relative. When they were unable to prevent the death of a relative, frustration and the idea that they could have prevented their death set in.

*“[ … ] I should have intervened, the symptoms were evident [ … ] I still think about how the diagnosis was delayed [niece with leukemia] and I am very frustrated because it was something that could have had a different outcome [ … ]”*. (Dr. 6)

On the other hand, approximately half of the interviewed residents preferred to isolate their caregiver roles, feeling overwhelmed by the situation, and preferred to simply act as a family member.

*"[...] and everyone is all over you: 'Did you go see him?' [...] And you think: 'What should I say, what should I do, how do I get through this?' I am not his doctor, so I cannot give a medical report [ … ] I am his relative [ … ]”.* (Dr. 4)

Several of the participants referred that experiences with relatives emotionally marked them and this was reflected by improved empathy with their subsequent patients. One of them mentioned that she distanced herself affectively from patients to avoid suffering and remain functional.

#### Concept of death in medical practice

To all participants, death was a daily event, but also an enemy to battle and conquer, leading some participants to always offer some potential cure despite its low probabilities of success.

*" [ … ] I don’t like to think that a patient doesn’t have a chance [...] I always offer something else that could be done [ … ]”*. (Dr. 3)

On the other hand, some interviewees considered that “there is always something that can be offered to patients”, such as palliative care, preferably in a timely manner.

### Category 2. Actions and reactions towards death

#### Recognition of imminent death in a patient

Most participants could recognize the conditions that signal imminent death, but they also mentioned that many factors, such as the patient’s socio-cultural context and attitudes, should be taken into consideration since they could modify the prognosis.

*"You know there is a body of knowledge in the medical literature, then you know how advanced the disease is and the chances that a patient has for healing [...] “.* (Dr. 2)

#### Communication of imminent death to patients and families

Almost all the interviewees acknowledged that communicating the imminence of death is one of the most complicated challenges in medical care, and even more so when applied to young patients*.* They state the importance of being honest with their patients, but by their accounts, they revealed that they are not completely clear with them. They avoid stating that death is imminent and prefer to offer convoluted medical explanations on disease and how difficult it is to predict death.

*"[...] Something I always tell those patients that ask me 'Am I going to die?' is 'Look, I would love to be the creator, to have a crystal ball so I could say Yes, the answer is yes, but I am human, I don’t know [ … ] I can’t give you that answer’ [ … ]”.* (Dr. 7)

Participants also justified this evasion by assuming that patients themselves are aware that they are going to die. They referred that “giving bad news” and the amount of information provided depend on each patient, but rather than exploring the patient’s expectations and needs, they tend to subjectively decide on a case-by-case basis. The fact that the family is frequently against sharing a terminal prognosis with the patient, significantly influences the physician-in-charge; almost all preferred not to communicate bad news at the relative’s request, although they personally considered that informing the patient is necessary.

*“[ … ] You can’t ignore the relatives who are asking you not to do it [deliver bad news], but it seems to me that the patient has the right to know [ … ] it makes me very angry that they are not told [ … ]”.* (Dr. 3)

This was in striking contrast with their personal conviction of being fully informed by their physicians, should they become ill. Finally, participants emphasized the importance of the context, whereby in private practice, communication is generally easier because they carry a lighter workload.

#### Reacting as a medical professional in the face of death

The situations causing participants the most feelings of frustration and sadness were the death of: (1) family members; (2) patients with high hopes of healing; (3) pediatric patients; (4) patients who have become emotionally close to them and (5) patients who reject the recommended treatments.

*“[ … ] I get really frustrated with those patients, then I get mad and say ‘Why don’t they want to try it if there is still something that can be done?' [ … ]”.* (Dr. 1)

For some participants, other situations affecting them were the deaths of patients with whom they identified or when they could relate with their loved ones, and in all cases, the first death they witnessed as doctors. On the other hand, death was more acceptable and less likely to burden them when: (1) they know from the beginning that there is no possible cure; (2) they have offered all the available therapeutic options; (3) death represents a solution to suffering and (4) patients have led a full life. In these cases, respondents embraced the concept of death as part of a vital process.

“*[ … ] I’ve done everything humanly possible for him [ … ] I don’t feel frustrated because since one first begins to treat patients like these, one is aware that treatments have limitations [ … ]”.* (Dr. 5).

Throughout their years of training, the death of their patients affected them less, since they had learned to emotionally distance themselves and delegate responsibilities.

#### Ways of coping with death

Interviewees used various coping mechanisms in the form of physical and emotional withdrawal when attempting to not become involved; they also established distances and raised barriers.

*“[...] I ​​do not want to relate too much with the patient [...] I frame a distance [...] is like my defense mechanism [ … ]"*. (Dr. 5)

However, this is not always possible, particularly when it comes to patients they have been treating for a long time*.* Participants asserted that it is necessary to get used to losing patients; sometimes, joking helps them to release the tension.

*“[...] With my peers sometimes we joke about things related to diseases [...] so everything you live daily doesn’t be so overwhelming [ … ]”.* (Dr. 6)

Becoming insensitive and blocking emotions helped some participants to cope and to move on*.* They recognized that these strategies were defense mechanisms reflecting that the death process actually does affect them. They tend to avoid talking about it because they feel it makes them seem weak, while still believing that their responsibility is to always be strong. Another way of coping mentioned by one resident was treating patients as if their diseases were not terminal.

*“[...] a very easy way out is to calmly establish limits and treat everyone as if they have a simple flu [...] I have not been able to do that [...] I've committed myself to the specialty [ … ]”*. (Dr. 1)

#### Support when dealing daily with death

Almost all participants initially seek support within their family unit, especially when one of its members is also a physician.

*“[...] I talk to my wife; she is a physician; we talk about medical issues [ … ]"*. (Dr. 4)

Respondents also seek support from co-workers, although usually, they avoid discussing death and the feelings it triggers. They avoid situations associated with death outside of the hospital. They all mentioned that moments of reflection were undoubtedly soothing as well as recreation, such as going to the movies, going out to dinner and being with family.

### Category 3. Training aspects to learn how to face death

#### The social representation of the physician figure

Participants considered that they should help patients within the limits established by the latter, without compromising their already undermined health, with full honesty and providing the greatest comfort possible.

*“[...] You study medicine to cure people [...] your obligation is to help them as much as they want [ … ]”*. (Dr. 5).

#### Specific training to face death as a physician

Most respondents referred never having coursed a class in medical school specifically focused on coping with death. In general, this training was empirically acquired.

*“[ … ] it’s something that is not learned, it is not something that is studied, it is something that is learned as you go [ … ]”.* Dr. 2

#### Models and anti-models

Participants stated the importance of observing how their teachers face death. The situations that have most marked them are the negative ones, from which they have learned what not to do. Few referred positive models that have taught them how to treat patients empathically and closely.

”*[ … ] He always said that they [patients] should be treated with respect and like we would want to be treated [ … ]”.* (Dr. 6)

#### Teaching others to face death

Respondents emphasized the importance of the education they provide to younger residents and students, but mentioned that it is not direct or formal but replaced by taking them on rounds so they can see “how it’s done”.

#### Self-perceived ability to cope with death

Residents commented that despite their poor training in “breaking bad news”, they believed that they were prepared to do so as a result of the empirical knowledge they acquired when they were responsible for providing this information as undergraduate students. However, they were convinced that this responsibility should lie in the treating physician’s hands. “It is obviously difficult to inform the family of a patient’s death when as a resident, you may not have even known him/her”.

*“[ … ] They never teach us how to deliver bad news [ … ] I have no idea if my method is good, if it is bad or if it is worse, but it is the one that has worked for me [ … ]”.* (Dr. 1)

#### Perceived needs to cope with death

Participants considered that EOL training should be compulsory for undergraduate students, particularly during their internship. On the one hand, they posited that when students are in medical school, they are unaware of the subject’s relevance since they have never faced death in medical practice. On the other hand, when dealing with terminally ill patients, they often have no time and are tired, and therefore, show no interest in adding another educational activity. Almost all agreed that EOL facts should be taught as short tutorials or seminars and that attending physicians should be more aware of their residents´ training needs. Some participants recognized the importance of psychotherapy for doctors who face death daily, but they were unwilling to actively seek external help.

*“[ … ] we say it jokingly, but we say that everyone in oncology has something wrong [ … ] I don’t know if it would be better if I had it [psychotherapy], or if I should look for it [ … ]”.*

(Dr. 7)

## Discussion

**Fig. 1:**
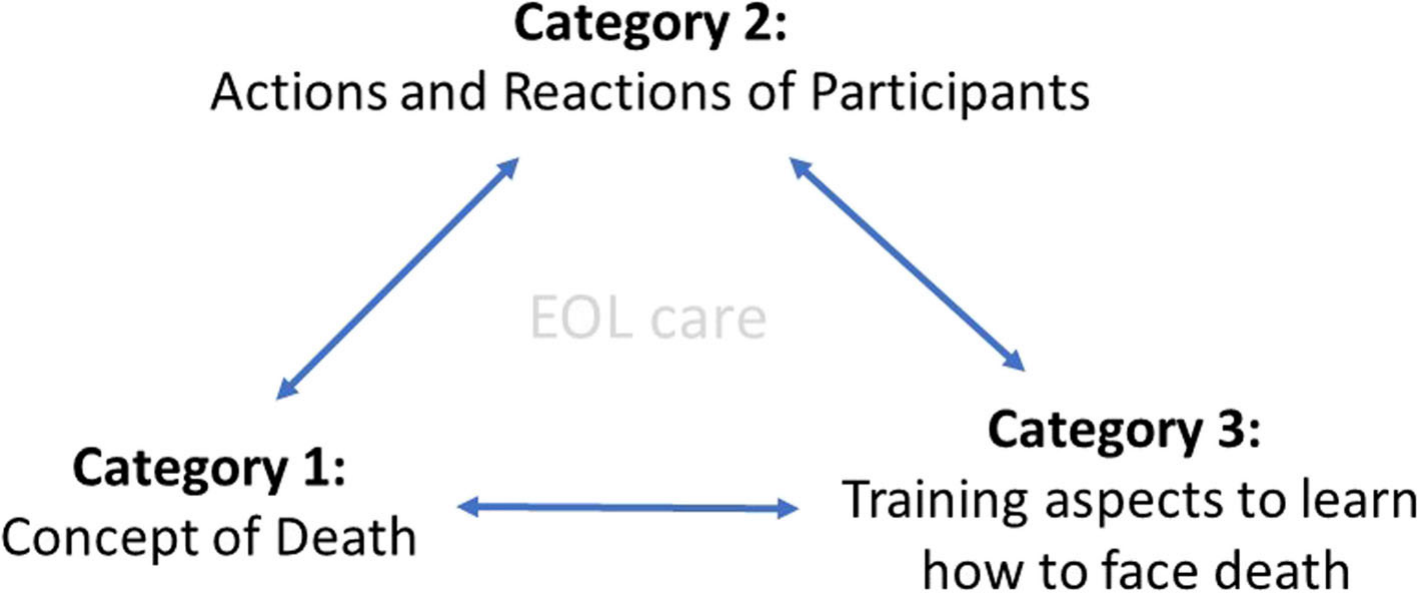
Interrelation of categories. EOL = end of life care

Considering that death occurs on a frequent basis in medical practice, and that it is frequently denied by inappropriately trained physicians, we explored through a qualitative design study, the experiences of oncology residents facing death-related situations in their practice. We also inquired on their personal concepts of death and their perceptions in terms of training since these latter two dimensions influence their behavior in practice, but at the same time, taking into account that these dimensions are modified by their experiences as doctors (Fig. 1).

Participants do not have a clear concept of death, as if they had not really reflected on it and also confirming their lack of training to deal with it. They view death as a transition, implying that life continues after death, an idea that is ultimately religious. This could be explained by the fact that even those who do not completely adopt the Catholic faith that prevails in our country, manifest its features in various areas of their lives. This persists in spite of publications that they read when they begin residency training, but their religious concept of death tends to progressively change into a biologically-based perception once they complete their formal education [26]. Respondents are not very conscious of how death affects them; although they state that they are not afraid of their own death, it does affect some when they identify with patients who are going to die, because as physicians, they also fear leaving their families helpless.

Aside from lacking a clear concept of death, participants find it very difficult to clearly and openly broach the subject with their patients, which in turn, compromises their patient-physician relationship. This may seem contradictory to the general perception that the Mexican culture provides a very familiar relationship with death, so as to handle it without conflict. Certainly, in Mexico, some people who conserve ancestral traditions and others, who regrettably are confronted daily with death due to extreme poverty or violence, handle it better [16]. For the rest of Mexicans, the supposed familiarity with death has been the result of political maneuvering last century, that in an attempt to awaken Mexico’s national identity, the representation of death became a theme, recurrently used by artists [27].

The residents in this study stressed the importance of individualizing the way in which bad news are conveyed, and considered that it is an ongoing and slow process that occurs over several contact periods and not a one-time assertion; some preferred to inspire hope in patients by emphasizing curative treatments instead of communicating dire prognoses and low probabilities of cure, as has been found in other studies [28]. The problem with fostering hope is that disappointment and frustration are worse when favorable outcomes never materialize. Regarding participants’ abilities to provide bad news, it seems that they consider themselves better prepared than they actually are; they are habitually ambiguous, evasive and assume that if patients do not ask, it is because they do not want to know.

Participants tended to agree with the family, should they request not to inform the patient on his prognosis, even if they personally considered that their patients should be well-informed. The influence of the family in this matter results from cultural idiosyncrasies. In Western countries, physicians prefer to respect the autonomy of their patients over the wishes of the family, while in Eastern countries, doctors need the approval of the family to communicate prognoses and decide on treatment options [29]. Although Mexico is a Western country, the family has a strong influence on the doctor-patient relationship, which explains why physicians hide information from their patients, if the family so wishes [17]. This contrasts with the honesty that a doctor would expect from his own physicians, should he find himself in the same situation.

Another paradigm that influences the participants is the socially accepted idea that physicians must somehow beat death and are required to always appear emotionally strong, as if sensitivity were a sign of weakness. Consequently, the most frequent emotional tools used by participants to cope with death were denial, insensitive behavior, and emotional detachment from patients. Granek et al. [30] considered that all these defense mechanisms were in part responsible for physician burnout, as well as the abuse of aggressive experimental treatments on their patients, with frequent undesirable side effects. Moreover, the fact that participants consider death as an enemy to overcome, is reflected in their actions and reactions, such as their persistence in offering treatments despite their low probabilities of success. At the same time, coping with their patient’s death appears to be less difficult once they have offered all available therapeutic alternatives, but more difficult if their patients reject any recommended therapies. These results are consistent with the literature in which current pervasive attempts to provide curative treatments, discomfort or inexperience with the concept and the realities of death, legal concerns, inadequate communication, uncertainty about the prognosis, request for additional treatment by the family, ignorance on the wishes of patients and the lack of availability of palliative treatments, were the main reasons given by physicians to administer futile treatments [31].

Our study detected that respondents considered their patients´ death more difficult if they had a close relationship with the patient, patients had unrealistic expectations of a cure, patients had prolonged suffering, patients had been treated for a long time period, physicians personally identified with the patient, unexpected deaths, or if there had been family disagreement on medical decisions. These conditions have proven to be responsible for prolonging physicians’ inner battles after their patients’ deaths [32]. These battles may be silent and unnoticed even by physicians themselves, affecting them personally, sometimes for years, until some event triggers its uncontrollable expression [33]. Nevertheless, participants considered that dealing with the death of pediatric patients was the most complicated scenario, a finding that had been previously reported by Granek [34].

To cope with death, our participants mentioned recreational activities as a source of emotional modulation. Other mechanisms often used by physicians are to consider death as a normal process and vouch to be more careful with subsequent patients. Searching for support within the family unit is another source of emotional relief and of utmost importance. Although residents attempt not to show their feelings with relatives, they do find support sharing their experiences (especially with a physician partner) and try to spend more time with family, a finding consistent with other studies in which physicians referred that speaking with team members (such as other physicians and nurses), friends and family members was their preferred coping strategy [35]. Participants in our study did not have private spaces in their hospitals to share and reflect on their experiences with a patient’s death, in clear contrast with hospital guidelines in other countries [35–39].

Although residents in this study recognized the importance of training to learn how to better deal with death, it seems that they are not fully invested in reaching out for more information, since they would not willingly reserve a time-space for self-training. While undergraduate students do have the time, participants considered that the former would be unaware of the subject’s relevance because they have yet to face death in clinical practice. However, there are studies that contradict this idea and show that medical students do benefit from early teaching of EOL care, not only to better treat dying patients, but patients in general [40, 41]. On the other hand, as mentioned by our respondents and in another study with resident participants, it was their personal experience with EOL care that taught them positive strategies they later applied in practice [42]. Additionally, although residents consider that psychotherapy would be helpful, they are not interested in seeking external professional help. The most useful recommendation they provided and which has recently been noted in another study [43], was that teaching-physicians in hospitals should be more aware of the residents´ training needs, instead of limiting their education to theoretical knowledge.

Probably, the main strength of this study is that it focuses on a group of young physicians only, so the results represent a specific subgroup of physicians, and the results can support actions and activities directly applicable to this group and help them refine their coping mechanisms. However, it is also a limitation because our findings cannot be extrapolated to other subgroups of physicians.

An important omission in this study is that the use of substances or other addictions to blunt the emotional response, was not explored as a possible coping mechanism, but has certainly been described by other authors [44].

Physicians are failing their patients at the EOL when providing them with false hopes and futile treatments instead of focusing on improving their quality of life, which could help them enjoy their last moments, surrounded by their loved ones and with the capacity to resolve or conclude pending issues [45]. Additionally, a physician suffering from a terminal illness has commented that in hospitals, doctors and patients live in two completely different worlds, whereby the medical world is relentlessly focused on curing diseases and complying with protocols, instead of trying to acknowledge the needs of their patients and alleviating the suffering of human beings. It seems that the lack of communication between both worlds has increased with the prevailing denial of death [46].

All physicians should be aware of how they are affected by death [47–49], and should focus on self-care [50], not only to provide better EOL care to their patients, but also for their own health, well-being and professional satisfaction. However, this is not only the responsibility of physicians; universities should include EOL care in their formal training curricula and promote quality continuing education during internship and medical residency. Medical institutions should also provide the means and spaces that could better help physicians to cope with the impact of death, in a private setting. It would also be fundamental to be keenly aware of the patients’ point of view in terms of their communication and information requests, as well as their emotional support needs.

## Conclusions

To explain the actions and reactions of oncology residents to death in their medical practice (Category 2) and to establish adequate strategies that will serve them in the future, we propose two dimensions that determine those actions and reactions: the personal concept of death that is initially acquired in the family, but is modified throughout training, and with the experiences of confronting death, both personally and professionally (Category 1); also, the formative aspects that include the social conception of physicians, and the theoretical and practical learning that they receive and provide (Category 3).

The residents in this study are forced to face death on a daily basis in their professional practice without the necessary training. This appears to impact them more than they are willing to accept, and they do not achieve their goals to manage terminal situations and death (especially communication) as well as they believe they do. Despite recognizing the need for further training and support to better cope with death, they are not actively seeking for specific information and support, maybe due to structural factors, individual reasons and/or the normalization of lack of training in death-related issues.

## Data Availability

This study is based on qualitative research. Interview material, voice recordings, transcriptions, and any raw data generated or analysed during this study are available on request.

## Abbreviations

COREQ: Consolidated Criteria for Reporting Qualitative Studies
EOL: End of life
NHATS: National Health and Aging Trends Study
